# Structural and Functional Characterization of the Enantiomers of the Antischistosomal Drug Oxamniquine

**DOI:** 10.1371/journal.pntd.0004132

**Published:** 2015-10-20

**Authors:** Alexander B. Taylor, Livia Pica-Mattoccia, Chiara M. Polcaro, Enrica Donati, Xiaohang Cao, Annalisa Basso, Alessandra Guidi, Anastasia R. Rugel, Stephen P. Holloway, Timothy J. C. Anderson, P. John Hart, Donato Cioli, Philip T. LoVerde

**Affiliations:** 1 Departments of Biochemistry, the University of Texas Health Science Center, San Antonio, Texas, United States of America; 2 X-ray Crystallography Core Laboratory, the University of Texas Health Science Center, San Antonio, Texas, United States of America; 3 Institute of Cell Biology and Neurobiology, CNR, Rome, Italy; 4 Institute of Chemical Methodologies, CNR, Rome, Italy; 5 Department of Pathology, the University of Texas Health Science Center, San Antonio, Texas, United States of America; 6 Texas Biomedical Research Institute, San Antonio, Texas, United States of America; 7 Department of Veterans Affairs, South Texas Veterans Health Care System, San Antonio, Texas, United States of America; McGill University, CANADA

## Abstract

**Background:**

For over two decades, a racemic mixture of oxamniquine (OXA) was administered to patients infected by *Schistosoma mansoni*, but whether one or both enantiomers exert antischistosomal activity was unknown. Recently, a ~30 kDa *S*. *m* *ansoni* sulfotransferase (SmSULT) was identified as the target of OXA action.

**Methodology/Principal Findings:**

Here, we separate the OXA enantiomers using chromatographic methods and assign their optical activities as dextrorotary [(+)-OXA] or levorotary [(-)-OXA]. Crystal structures of the parasite enzyme in complex with optically pure (+)-OXA and (-)-OXA) reveal their absolute configurations as *S*- and *R*-, respectively. When tested *in vitro*, *S*-OXA demonstrated the bulk of schistosomicidal activity, while *R*-OXA had antischistosomal effects when present at relatively high concentrations. Crystal structures *R*-OXA•SmSULT and *S*-OXA•SmSULT complexes reveal similarities in the modes of OXA binding, but only the *S*-OXA enantiomer is observed in the structure of the enzyme exposed to racemic OXA.

**Conclusions/Significance:**

Together the data suggest the higher schistosomicidal activity of *S*-OXA is correlated with its ability to outcompete *R*-OXA binding the sulfotransferase active site. These findings have important implications for the design, syntheses, and dosing of new OXA-based antischistosomal compounds.

## Author Summary

Schistosomes, parasites that cause the disease schistosomiasis in humans, are blood flukes that infect an estimated 200 million people in 76 countries. Control of schistosomiasis is currently based on repeated doses of the drug praziquantel (PZQ). Parasites showing reduced susceptibility to PZQ have been recovered from patients that failed PZQ treatment and have been obtained by experimental selection. New anti-schistosomal drugs are therefore needed that can be used with PZQ to minimize the probability of resistance. The older anti-schistosomal drug oxamniquine (OXA) has an excellent efficacy and safety record but is only active against one of the three species infecting humans. Recently, a combination of genetic and structural analyses resulted in the determination of the structure of OXA in complex with its target enzyme in the parasite, providing opportunity for structure-guided modifications of OXA to make it more effective against all three schistosome species. Synthesis of OXA results in a racemic mixture. Here, we isolate OXA enantiomers and find that one is more effective than the other at killing schistosomes. Crystal structures of both OXA enantiomers bound to the target enzyme suggest a molecular basis for this observation that should be considered in ongoing and future OXA-based drug design efforts.

## Introduction

For more than 25 years, the mainstay of treatment for *Schistosoma mansoni* infections in Brazil was the drug oxamniquine (OXA, (*RS*)-1,2,3,4-tetrahydro-2- isopropylaminomethyl-7-nitro-6-quinolylmethanol)[1,2]. OXA is species-specific, killing *S*. *mansoni* (67 million cases worldwide) but not other schistosome species in Africa (*S*. *haematobium*, 119 million cases) or in SE Asia (*S*. *japonicum*, 1 million cases)[3,4]. OXA is no longer manufactured because the drug praziquantel, which is effective against all schistosome species, is now available at a reasonable price due to the expiration of its patent.

The mode of action of OXA was recently elucidated[5]. As predicted by Pica-Mattoccia *et al*. [6], OXA is a prodrug that is taken up by the parasite and sulfonated by an endogenous sulfotransferase (SmSULT, GenBank AHB62207.1, UniProt V9PWX8) in the presence of 3’phosphoadenosine 5’phosphosulfate (PAPS). The resulting sulfate ester of OXA is an unstable species that spontaneously decays to form a reactive electrophilic product capable of alkylating DNA, proteins and other macromolecules. The ensuing disruption of synthetic and metabolic cellular functions eventually leads to parasite death[5,7].

**Fig 1:**
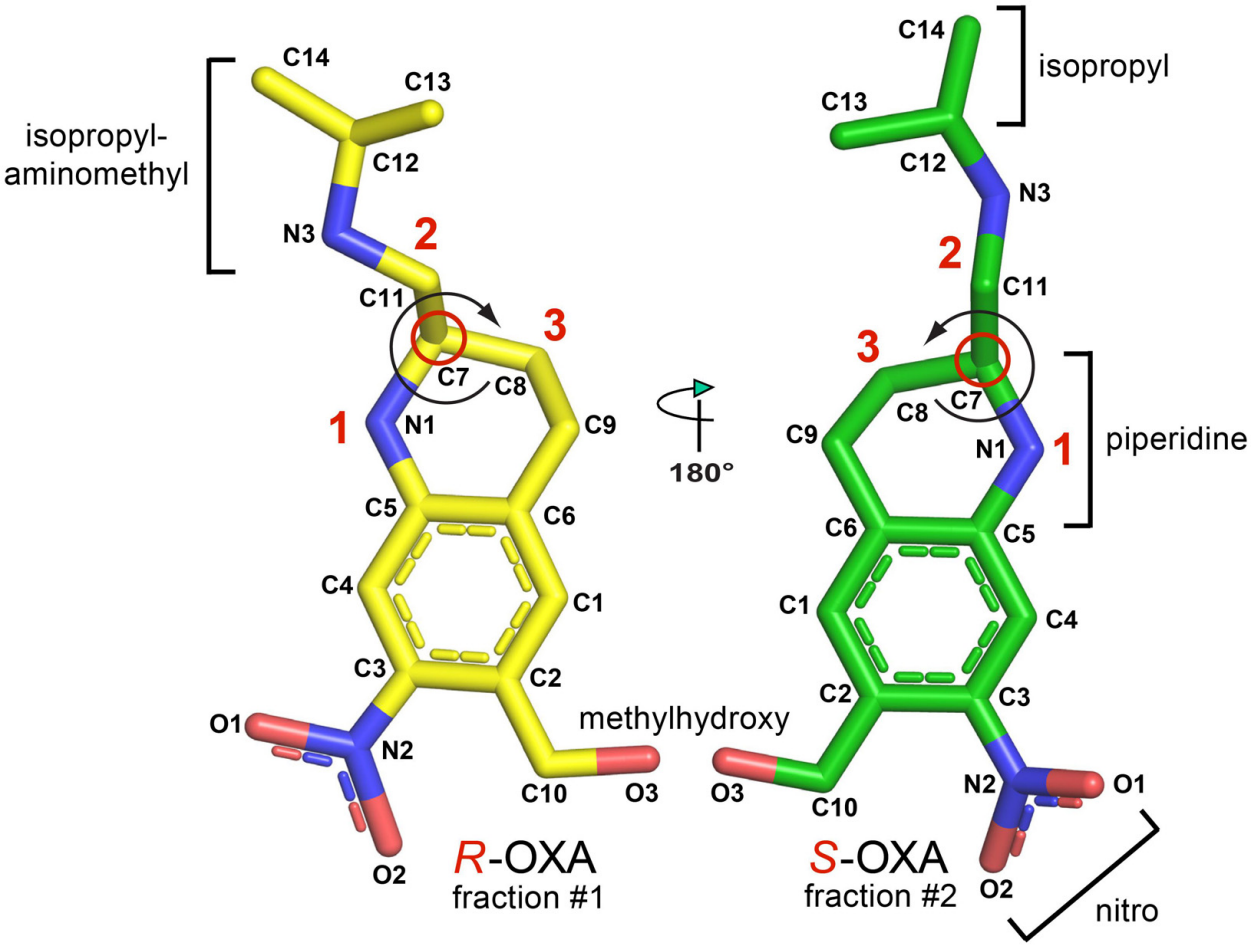
*R*-oxamniquine and *S*-oxamniquine indicating the functional groups and the chiral carbons (red circles). The absolute configurations are determined by the substituents coming off of the chiral carbon atom using the Cahn-Ingold-Prelog rules. The molecules are rotated such that the lowest priority substituent (H-atom) is in the back of the chiral carbon, facing away from the viewer. Curves are drawn from substituents 1 to 2 to 3 substituent. If two substituents have the same immediate substituent atom, evaluate atoms progressively further away from the chiral center until a difference is found. If the curve is clockwise, the stereocenter is of *R*-configuration. If the curve is counterclockwise, the stereocenter is of *S*-confguration.

OXA possesses one asymmetric carbon atom and its two enantiomers are both present in the marketed drug (Fig 1). The structure of SmSULT with bound OXA and depleted co-factor PAP was determined previously at a resolution of 1.75 Å [pdb code 4MUB, [5]. Although the crystals were soaked with racemic OXA, the structure revealed only *S*-OXA in the central cavity of the L-shaped, predominantly α-helical enzyme with its hydroxyl group (the target of sulfonation) centered at the end of a shaft running from the surface of the molecule. The relative positions of the accepting OXA and donating PAPS groups are entirely consistent with the formation of a sulfonated OXA hydroxyl group (sulfate ester of OXA).

Previous studies have succeeded in separating the enantiomers, but have not provided information about their absolute configurations or relative antischistosomal properties [8–10]. The question of which or if both enantiomers are active, and which or if both can occupy the binding pocket of SmSULT is addressed in this report.

## Methods

### Source of materials

Racemic OXA was a gift from Dr. D. Buggey (Pfizer Ltd.).

### HPLC chiral separation

The separation of OXA enantiomers was carried out by HPLC on a modified cellulose chiral stationary phase (Chiracell OD-H, Daicel Chemical Industry) 4.6 mm i.d. x 150 mm, eluted at 1 mL/min with a mixture of 5% isopropanol in n-hexane (HPLC grade, Carlo Erba, Italy), added with 0.1% dimethylamine (Aldrich, 99.5% purified by distillation). The column consists of Cellulose tris (3,5-dimethylphenylcarbamate) physically coated on microparticulate silica gel. This chiral selector is versatile and it shows a particularly good selectivity towards aromatic compounds with substituents containing N or O atoms. Dimethylamine was added to the eluent to prevent the ionization of OXA amino groups, but it was removed immediately after fraction collection, to avoid degradation of purified OXA isomers. The chromatogram was obtained at 254 nm. Fifty μL of a racemate solution (1 mg/mL) in *n*-hexane:isopropanol (1:1) were injected into the column and the separated enantiomer peaks were collected. The racemate solution was stored at –20°C. In order to obtain sufficient material, the separation was repeated several times and the collected eluates were immediately evaporated to dryness under vacuum, in order to remove the dimethylamine. The pool of evaporated fractions corresponding to the two enantiomers were re-dissolved in 1 mL of *n*-hexane:isopropanol and 10 μL analyzed by HPLC in the chromatographic system described above. This allowed control of purity and quantitative determination by comparison with a calibration curve obtained with a known amount of racemate.

### Capillary zone electrophoresis (CZE) separation

A solution of 0.58 mg/mL of OXA racemate was prepared in CH_3_OH:H_2_O (60:40) and then diluted with the running buffer (50 mM pH 3 phosphate) to obtain a 35 μg/mL solution. The CZE separation was performed as described by Abushoffa & Clark [8]. The background electrolyte consisted of running buffer with 1 mM heparin as a chiral selector. Separation was performed in a 62 cm, 75 μm i.d. capillary tube at 30°C, with an applied voltage of 20 kV. Samples were hydrodynamically injected (50 mbar, 5 sec).

Fifty μL of enantiomer #1 (*i*.*e*. the first eluting compound in HPLC) was evaporated to dryness with a N_2_ flux and subsequently dissolved in 20 μL of 5% methanol in running buffer. Ten μL of this enantiomer #1 solution was added to 600 μL of racemate (35 μg/mL) and the mixture was analyzed by CZE under the same conditions described above for the racemate.

### Protein crystallization, structure determination and refinement

The sulfotransferase from *S*. *mansoni* (SmSULT) was crystallized as described previously [5]. The depleted co-factor PAP (3’phosphoadenosine 5’phosphate) was added to achieve a 4 to 1 stoichiometric ratio over protein and incubated for one hour on ice prior to crystallization. Freshly grown crystals (4–8 days post-setup) were soaked overnight in saturating conditions of each purified OXA enantiomer and flash-cooled in liquid nitrogen prior to data collection. All diffraction data were measured at the Advanced Photon Source NE-CAT beamline 24-ID-C and integrated and scaled using the program XDS [11]. Structures of the OXA enantiomer•SmSULT•PAP complexes were isomorphous with the published SmSULT structure (Protein Data Bank entry 4MUA) and the OXA enantiomers were built into difference electron density with coefficients *m*F_o_-*D*F_c_ [12]. Model coordinates were refined using the PHENIX program suite [13], including simulated annealing with torsion angle dynamics, alternating with manual model adjustment using the program COOT [14]. Figures depicting protein and OXA structure were created using the program PyMOL [15]. Coordinates and structure factors have been deposited in the Protein Data Bank [16] under accession codes 5BYJ and 5BYK.

### Ethics

For the Institute of Cell Biology and Neurobiology, experimental protocols involving the use of animals were reviewed and approved by the Public Veterinary Health Department of the Italian Ministry of Health (Authorization N. 25/2014-PR). For the University of Texas Health Science Center (S2 Fig legend), the use of animals in his study was approved by the University of Texas Health Science Center IACUC (Protocol 11087x) that adheres to the NIH Animal Care and Use guidelines.

### Parasite maintenance

A Puerto Rican strain of *S*. *mansoni* that has been maintained in the laboratory for several decades was used throughout this study. An albino strain of *Biomphalaria glabrata* served as the intermediate host, while CD1 female albino mice (Harlan, Italy) were used for the mammalian stages. Unisexual infections were obtained by exposing snails to a single miracidium and then sexing the emerging cercariae by PCR using female-specific W1 primers [17].

### Drug assay

Mice infected by tail immersion with 160 male cercariae were perfused ≥7 weeks later and the worms obtained were used for drug assays. Eight to 13 male worms were distributed in tissue culture dishes (3.5 cm) in Dulbecco modified Minimum Eagle’s Medium (bicarbonate buffered) supplemented with 10% fetal calf serum, 100 U/mL penicillin, 100 μg/mL streptomycin and 0.5 μg/mL amphotericin B. Cultures were kept at 37°C in an atmosphere of 5% CO_2_ in air and were observed daily under a Leica MZ12.5 stereomicroscope. Male worms were used as they are more sensitive to the effects of oxamniquine then are female worms [18]. Parasites were exposed to racemic OXA or its purified enantiomers for 30 min and subsequently washed three times and transferred to new dishes containing drug-free medium. At the end of the observation period (2 weeks at high doses; 3 weeks at low doses), worms were classified on the basis of various vitality indicators, as: *normal* (similar to untreated controls) and assigned score 100; *slow* (decreased motility and slight morphological changes), score 60; *moribund* (only tiny movements, marked morphological changes, opaque appearance), score 30; *dead* (no movement, severe morphological changes, dark appearance), score 0. The number of worms in each category was recorded. The scores of all worms were added, divided by the number of worms present in the dish and reported as average scores.

## Results

### Isolation and identification of enantiomers

**Fig 2:**
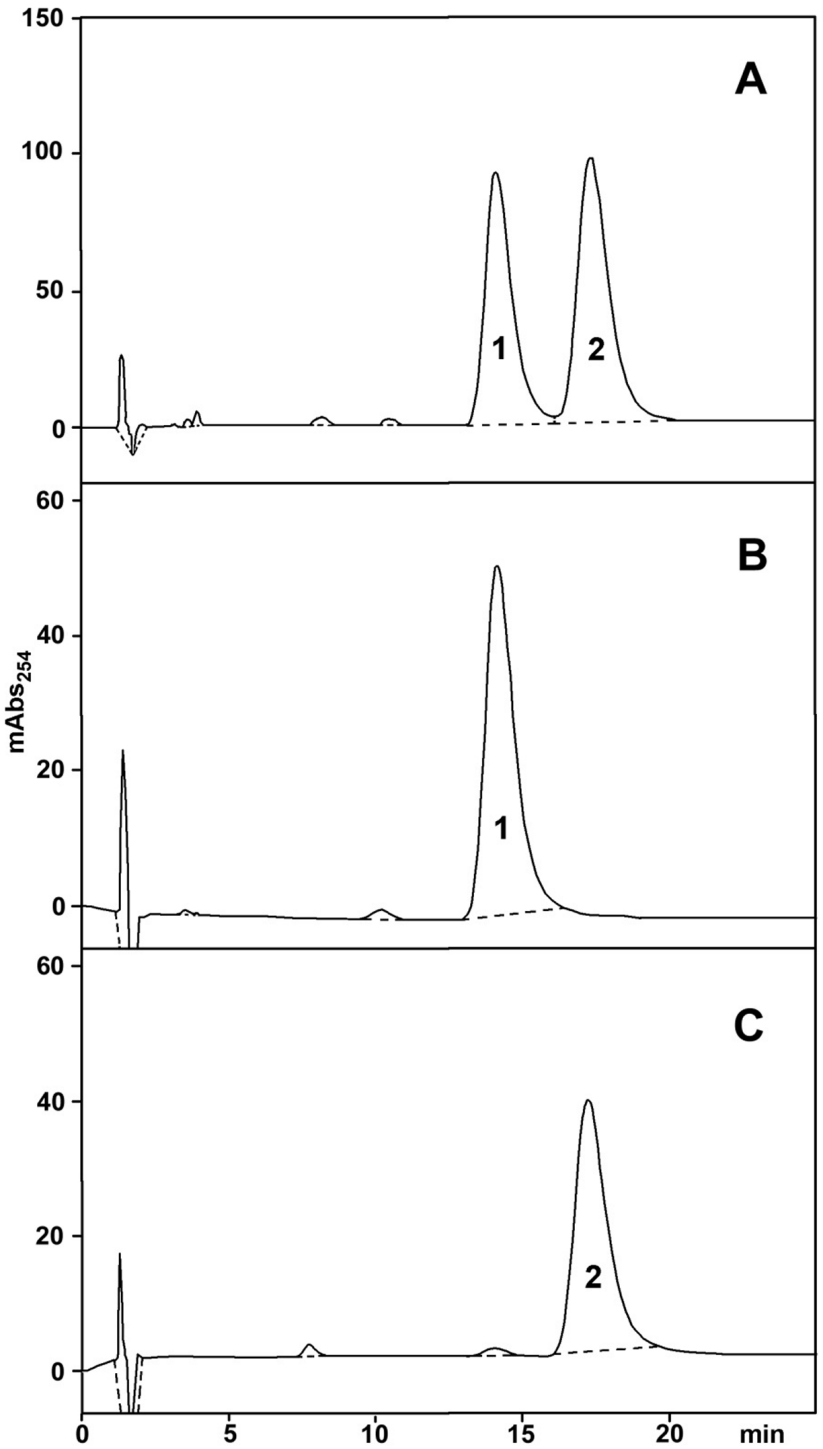
**A) Separation of oxamniquine stereoisomers by HPLC on a chiral column.** B) HPLC purity control on the same column of a pool of fractions # 1 obtained from several runs like the one depicted in A. C) HPLC purity control on the same column of a pool of fractions # 2 obtained from several runs like the one depicted in A.

The enantiomers of OXA were separated by semi-preparative HPLC on a chiral column and the results are illustrated in Fig 2A. Two major peaks were present, practically with baseline separation, and were provisionally labeled as #1 and #2. The area under the curve was essentially the same for the two peaks, consistent with the enantiomers being present in equal amounts. Since the quantity of compounds obtained from a single separation was limited, fractions #1 and #2 from several runs were pooled, respectively, and re-applied to the same column to check purity and to estimate quantity. As shown in Fig 2B and 2C, the separated enantiomers were reasonably pure and their total amounts were estimated to be about 200 μg for each enantiomer.

**Fig 3:**
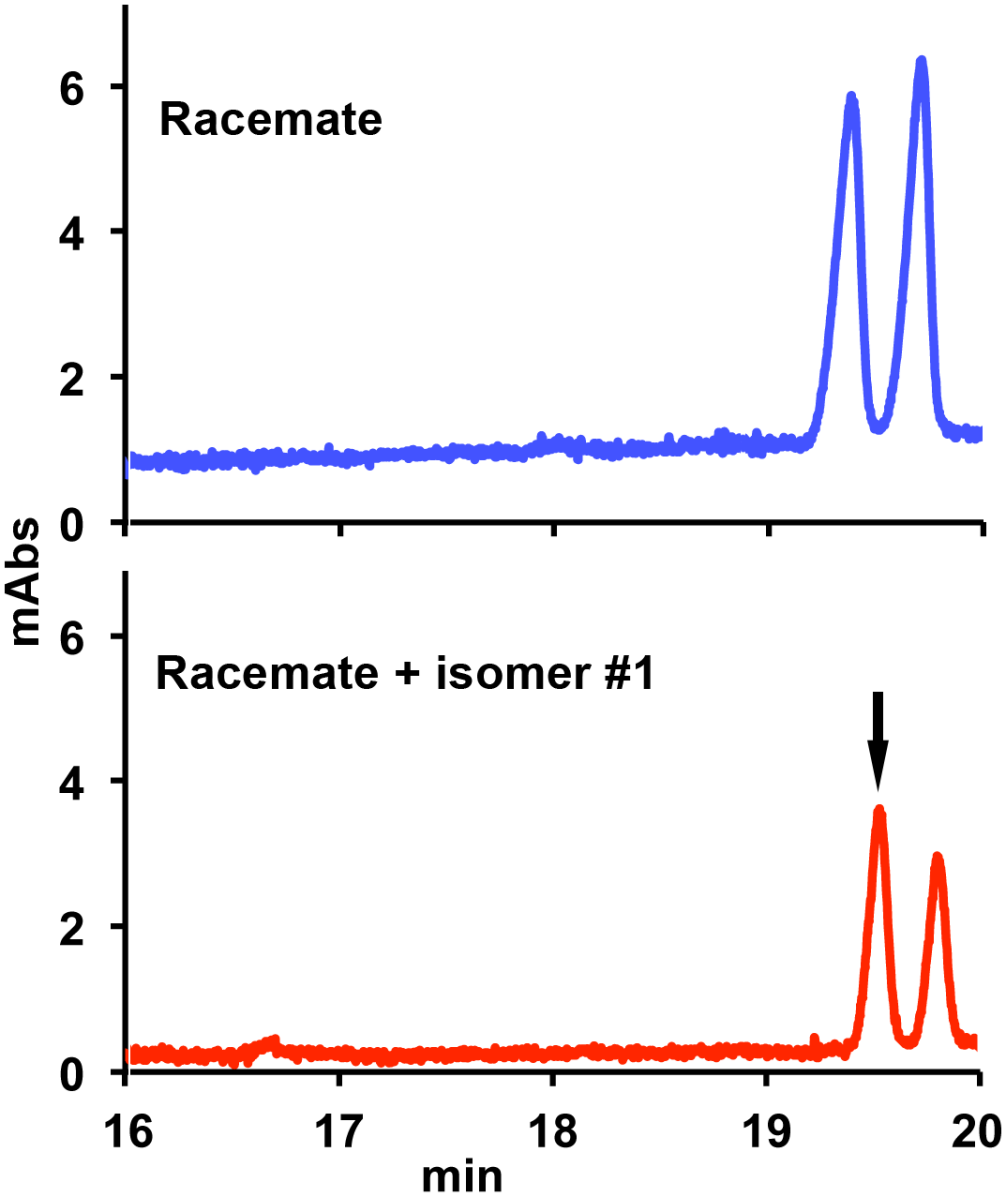
Separation of oxamniquine stereoisomers by capillary zone electrophoresis. Top: profile of racemic oxamniquine alone. Bottom: profile of racemic oxamniquine spiked with isomer #1 (arrow) purified by HPLC.

The small amount of material required by CZE prompted us to use this technique in order to assign the optical activity of each enantiomer as either dextro- or levo-rotatory. In a previous separation by CZE, Abushoffa & Clark [8] showed that the OXA levorotatory (–) enantiomer has a higher electrophoretic mobility than the dextrorotatory (+) one. A racemate solution spiked with enantiomer #1, obtained from the chromatographic separation, was then analyzed by CZE as previously described. Since compound #1 co-migrated with the faster peak of the racemic mixture, it was identified with the levorotatory (–) enantiomer (Fig 3).

### Crystal structures of SmSULT/OXA enantiomer complexes

**Fig 4:**
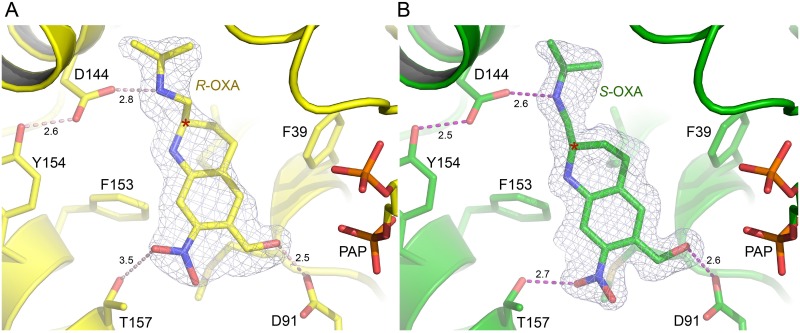
Oxamniquine enantiomers bound to the SmSULT active site. A) 1.8 Å electron density calculated with coefficients 2*m*F_o_-*D*F_c_ contoured at 1.0σ superimposed on the refined structure of the *R*-OXA•SmSULT•PAP complex. Numbers represent H-bond lengths in Å. B) 1.3 Å electron density calculated with coefficients 2*m*F_o_-*D*F_c_ contoured at 1.0σ superimposed on the structure of the *S*-OXA•SmSULT•PAP complex. Note: some structural elements have been removed for clarity.

In our previous work, SmSULT crystals were soaked with racemic OXA prepared for medicinal use [5]. Although the preparation contained an approximate 1:1 mixture of enantiomers, only one (*S*-OXA, see below) was observed in the crystal structure. In the present study, single SmSULT crystals were soaked with purified preparations of either *R*- or *S*-OXA enantiomers. Data collection and refinement statistics for the two new structures determined in this study are shown in Table 1. The OXA enantiomers bind in similar overall orientations (Fig 4A and 4B). The crystal structures clearly reveal the chirality of each compound and identify the (–)-enantiomer (peak #1) as *R*-OXA and the (+)-enantiomer (peak #2) as *S*-OXA [19]. The positions and orientations of amino acid residues contacting the two enantiomers of OXA are virtually unchanged in the two structures and it is the relative positions of the two enantiomers of OXA and the water structure that adjust to best accommodate each enantiomer in the SmSULT binding cavity.

**Table 1:** X-ray diffraction data collection and refinement statistics.

	SmSULT + *R*-OXA	SmSULT + *S*-OXA
PDB code	5BYJ	5BYK
**Data collection**		
Space group	*P*2_1_2_1_2	*P*2_1_2_1_2
Cell dimensions		
*a*, *b*, *c* (Å)	140.5, 39.2, 54.0	140.6, 39.4, 54.0
α, β, γ (°)	90, 90, 90	90, 90, 90
Wavelength (Å)	0.9792	0.9792
Resolution (Å)	42.82–1.80 (1.90–1.80)*	54.00–1.28 (1.35–1.28)
*R*_sym_	0.054 (0.652)	0.052 (0.693)
*R*_pim_	0.027 (0.359)	0.028 (0.365)
*I*/σ*I*	15.3 (2.1)	14.9 (2.1)
Completeness (%)	98.7 (98.5)	97.8 (96.7)
Redundancy	4.3 (4.0)	4.3 (4.2)
Wilson value	29.1	16.7
**Refinement**		
Resolution (Å)	42.82–1.80	50.41–1.28
No. reflections	27,971	76,328
*R*_work/_ *R*_free_	0.173/0.204	0.152 (0.171)
No. atoms		
Protein	2,105	2,100
Ligand/ion	47 (1 PAP, 1 OXA)^†^	57 (1 PAP, 1 OXA, 1 DMA, 1 Na^+^)
Water	217	334
B-factors (Å^2^)		
Protein	31.5	21.6
Ligand	32.3	21.6
Solvent	39.5	35.8
R.m.s deviations		
Bond lengths (Å)	0.007	0.007
Bond angles (°)	1.054	1.105
Ramachandran statistics—favored, allowed, outliers (%)	97.2, 2.8, 0.0	98.1, 1.9, 0.0

*Highest resolution shell is shown in parentheses.

^†^Ligand abbreviations: DMA—dimethylarsenic cysteine (cysteine modified by cacodylate present in the crystallization buffer), OXA—oxamniquine, PAP—adenosine-3'-5'-diphosphate

Some of the ordered water structure in contact with OXA is preserved between enantiomer complexes including water molecules that form hydrogen bonds to the hydroxy moiety, the amine in the isopropylaminomethyl moiety, or make a van der Waals contact to the isopropyl moiety (S1 Fig). A water molecule unique to the *R*-OXA complex is observed in hydrogen bonding distance to the piperidine amine. The orientation of *S*-OXA prevents a water molecule from occupying this same position, but two additional water molecules unique to the *S*-OXA complex are observed nearby within van der Waals contact distances (S1 Fig).

**Fig 5:**
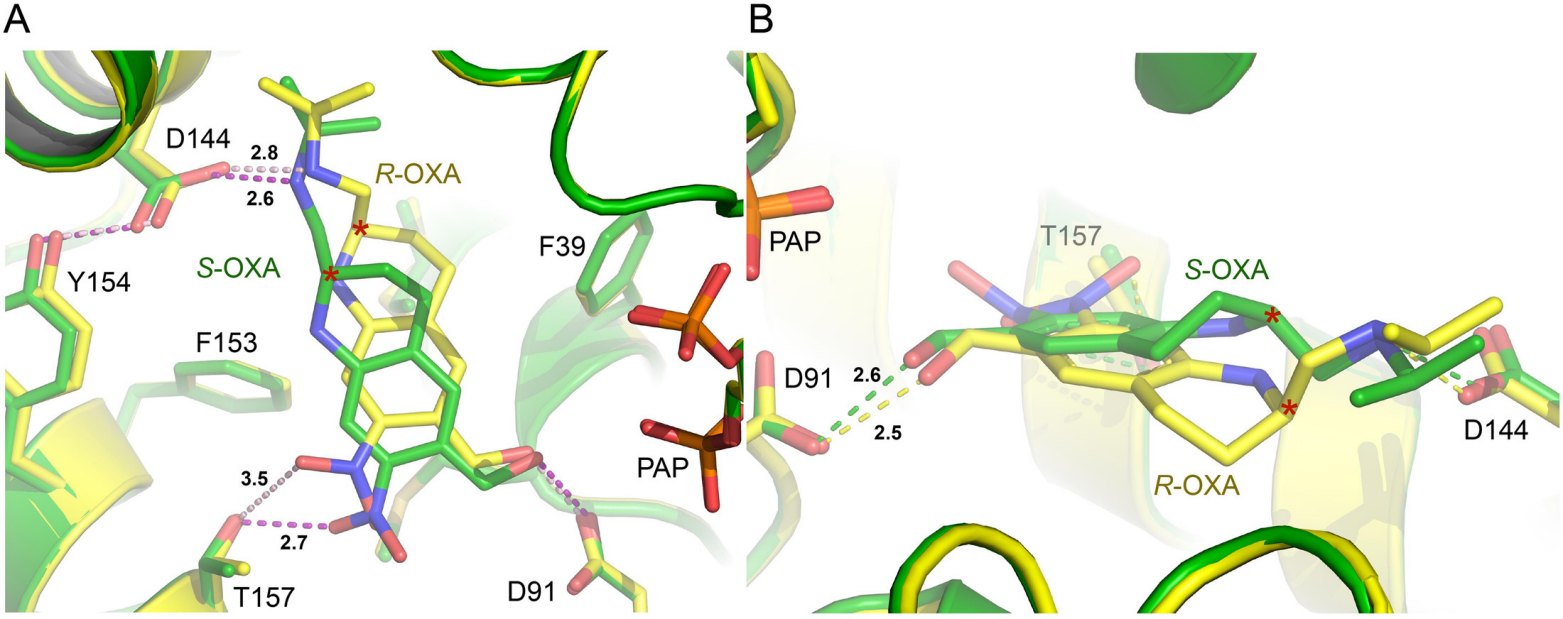
**A) Superimposed structures of *R*- and *S*-OXA complex structures in yellow and green, respectively.** Hydrogen bonding distances are indicated as dashed lines with distances in Å. B) Alternate view of superimposed enantiomer complex structures highlighting the pucker of the piperidine ring. The chiral carbon is marked (*). Note: some structural elements have been removed for clarity.

The isopropylaminomethyl and piperidine moieties (Figs 1 and 5A and 5B) of the OXA enantiomers are observed in different configurations. The terminal methyl groups of the isopropyl moiety in each enantiomer orient in the same direction and the adjacent secondary amino groups are both observed in hydrogen bonding distance to Asp144 (Figs 4 and 5A). However, the chiral carbon atoms of the piperidine moieties joining the isopropylaminomethyl moieties in each enantiomer force the linked methyl groups to orient in opposite directions (Fig 5B). Additionally, the piperidine rings bend in opposite directions at the carbon position next to the chiral carbon, relative to the plane of the rings thereby adopting opposite ring puckers (Fig 5B).

While the OXA nitro and hydroxymethyl substituent groups are rotated with respect to each other about an axis perpendicular to the aromatic ring, the accepting hydroxyl groups in both enantiomers essentially overlap and are therefore both in position to hydrogen bond to the side chain of Asp91 (Fig 5A). The nitro moiety of *S-*OXA maintains a hydrogen bond (2.7Å) with Thr157 as previously observed in the SmSULT complex structure determined in the presence of the racemic mixture. The nitro moiety of *R*-OXA is significantly rotated relative to that in S-OXA and positioned oriented at a greater distance from Thr157 (3.5Å) suggesting a comparatively weak hydrogen bond (Fig 5A). Overall, the ring structure of *R*-OXA rotates ~10 degrees about an axis perpendicular to the plane of the ring relative to *S*-OXA.

### Antischistosomal properties

The separated OXA enantiomers, together with the racemic mixture, were tested *in vitro* for their relative schistosomicidal properties. In these experiments, the concentration of racemate was double that of the enantiomers, under the assumption that only one of the two enantiomers might be active, so that the racemate would contain only 50% of active substance. In order to take into account as much as possible the variety of effects exerted on schistosomes by the different compounds, we preferred to adopt a vitality scoring system rather than a simple live/dead classification. Four different experiments were performed at the Institute of Cell Biology and Neurobiology, two of them at low doses (upper half of Table 2) and two at high doses (lower half of Table 2). The results of the two experiments at each dose range were pooled and averaged.

**Table 2:** Effects of OXA and its separate enantiomers on *S*. *mansoni* kept *in vitro*. Morphological appeareance and movements of worms were recorded two or three weeks after drug exposure, for high and low doses respectively.

		Number of worms in each condition				
Treatment	μg/mL	Normal *(Score 100)*	Slow *(Score 60)*	Moribund *(Score 30)*	Dead *(Score 0)*	Average score
Medium + DMSO	0	20				100.0
OXA racemate	8		2	8	15	14.4
*R*-OXA	4	16	2	2		89.0
*S*-OXA	4		5	15	3	32.6
Medium + DMSO	0	24				100.0
OXA racemate	40			6	18	7.5
*R*-OXA	20			17	6	22.2
*S*-OXA	20		1	10	13	15.0

In the set of experiments carried out at low drug concentration, OXA racemate at 8 μg/mL reduced worm vitality to 14.4% of controls, while *S*-OXA (at half that concentration) was less effective, decreasing vitality to 32.6% of controls. *R*-OXA, on the other hand, had only a very modest effect, lowering worm vitality to about 89% of controls. Thus, under these conditions, *S*-OXA had about 3X the activity of *R*-OXA.

When 5X higher drug concentrations were used (lower half of Table 2), the effect on worms was obviously more pronounced for all compounds, and even *R*-OXA displayed a sizeable activity (bringing worm vitality down to 22.2% of controls) (see S1–S4 Videos). In an independent set of experiments conducted at UTHSCSA, using a different schistosome strain and minor methodological variations, adult schistosome male worms were treated with 40 μg/mL of racemate OXA and 20 μg/mL of each enantiomer. Under these conditions, *S*-OXA clearly showed a much higher activity than *R*-OXA, thus confirming the above results (S2 Fig).

Taken together, these results suggest that the bulk of antischistosomal activity is exerted by *S*-OXA, but even *R*-OXA can have antischistosomal effects when present at high concentrations. This is confirmed by the fact that the racemate at 8 μg/mL is more effective than *S*-OXA enantiomer at half the concentration, possibly due to a contribution of *R*-OXA to the overall activity. In order to rule out the possibility that the separated enantiomers might induce some non-specific toxicity, we had preliminarily ascertained that the OXA-resistant schistosome strain HR [5] was completely unaffected by these substances.

## Discussion

The separation of the two OXA enantiomers had been previously described using either chromatographic or electrophoretic approaches [8–10], but the relative contributions of each enantiomer to antischistosomal activity and their individual involvement in the molecular mechanism of action had not been addressed. Data presented here show that the *S*-(+)-enantiomer is responsible for the majority of the antischistosomal activity, while the *R*-(–)-enantiomer is capable of exerting a moderate activity that is best detected when present alone and at relatively high concentration. Indeed, the crystal structures reveal both enantiomers bind similarly in the central cavity of SmSULT, although when crystals are exposed to a racemic mixture of OXA electron density for only *S*-OXA is observed. The structures of both enantiomer complexes with SmSULT reveal that the positions of amino acid residues surrounding OXA in the central cavity do not vary significantly. Instead, it is the orientation and configuration of the OXA enantiomers that varies in order to occupy the central cavity. Although *R*- and *S*-OXA occupy much of the same space in the cavity, the positions of the piperidine moiety and its isopropylaminomethyl substituent demonstrate the most variation due to influence of the chiral carbon while preserving the positioning of the methylhydroxy group which is the target of modification by the sulfotransferase.

The combined results of these molecular and biological analyses suggest that when schistosomes are exposed to the racemic OXA mixture, the activating enzyme SmSULT preferentially binds and sulfonates the *S*-OXA, which has an overall better steric fit for the central cavity of the protein. The piperidine rings in the enantiomers show opposite puckers adjacent to the chiral carbon, but the adjoining isopropylaminomethyl groups of *R*- and *S*-OXA occupy similar positions in the binding pocket (Fig 3). *R*- and *S*-OXA also maintain similar hydrogen bonding distances (2.8 and 2.6 Å, respectively) between the isopropylamino group and the Asp144 side chain Oδ. However, a more favorable hydrogen bond is formed by Thr157 to the nitro group of *S*-OXA (2.7 Å) compared to *R*-OXA (3.5 Å). Thus, the *S*-OXA enantiomer may out-compete *R*-OXA due to a more favorable energy of binding in the racemic mixture. Kinetic data, which we are attempting to generate, may help address the issue of of why S is better than R in terms of activity.

As with many other drugs, it would be desirable that stereochemically homogeneous compounds be employed as antischistosomal agents. Our ongoing efforts to generate novel wide spectrum OXA derivatives will definitely take this option into account.

## Supporting Information

S1 Fig Water structure in contact with oxamniquine enantiomers. Superimposed structures of *R*- and *S*-OXA complex structures in green and yellow, respectively.
(DOCX)

S2 Fig Assay for efficacy of racemic OXA and its enantiomers. Ten adult male worms per well. Three wells per drug. Treated for 45min with 40 μg/ml of OXA and 20 μg/ml of each enantiomer or DMSO equivalent.
(DOCX)

S1 Video Video showing the movement of adult male *Schistosoma mansoni* worms treated with the diluent (DMSO). (AVI)

S2 Video Video showing the movement of adult male *Schistosoma mansoni* worms treated with 8 μg of Oxamniquine. (AVI)

S3 Video Video showing the movement of adult male *Schistosoma mansoni* worms treated with 4 μg of S-enantiomer (Fraction 1 in Fig 2) of Oxamniquine. (AVI)

S4 Video Video showing the movement of adult male *Schistosoma mansoni* worms treated with 4 μg of R-enantiomer (Fraction 2 in Fig 2) of Oxamniquine. (AVI)

## References

[1] Coura JR, Amaral RS (2004) Epidemiological and control aspects of schistosomiasis in Brazilian endemic areas. Mem Inst Oswaldo Cruz 99: 13–19. 15486629 10.1590/s0074-02762004000900003

[2] Committee WHOE (2002) Prevention and control of schistosomiasis and soil-transmitted helminthiasis. World Health Organ Tech Rep Ser 912: i–vi, 1–57, back cover. 12592987

[3] Foster R, Cheetham BL, King DF (1973) Studies with Schistosomicide Oxamniquine (Uk-4271) .2. Activity in Primates. Transactions of the Royal Society of Tropical Medicine and Hygiene 67: 685–693. 4205106 10.1016/0035-9203(73)90039-4

[4] PicaMattoccia L, Novi A, Cioli D (1997) Enzymatic basis for the lack of oxamniquine activity in Schistosoma haematobium infections. Parasitology Research 83: 687–689. 9272559 10.1007/s004360050320

[5] Valentim CLL, Cioli D, Chevalier FD, Cao XH, Taylor AB, et al (2013) Genetic and Molecular Basis of Drug Resistance and Species-Specific Drug Action in Schistosome Parasites. Science 342: 1385–1389. doi: 10.1126/science.1243106 24263136 10.1126/science.1243106 PMC4136436

[6] Pica-Mattoccia L, Carlini D, Guidi A, Cimica V, Vigorosi F, et al (2006) The schistosome enzyme that activates oxamniquine has the characteristics of a sulfotransferase. Mem Inst Oswaldo Cruz 101 Suppl 1: 307–312. 17308787 10.1590/s0074-02762006000900048

[7] Cioli D, Pica-Mattoccia L, Rosenberg S, Archer S (1985) Evidence for the mode of antischistosomal action of hycanthone. Life Sci 37: 161–167. 4010473 10.1016/0024-3205(85)90419-9

[8] Abushoffa AM, Clark BJ (1995) Resolution of the enantiomers of oxamniquine by capillary electrophoresis and high-performance liquid chromatography with cyclodextrins and heparin as chiral selectors. J Chromatogr A 700: 51–58. 7767464 10.1016/0021-9673(95)00111-y

[9] Noctor TAG, Fell AF, Kaye B (1990) High-Performance Liquid-Chromatographic Resolution of Oxamniquine Enantiomers—Application to Invitro Metabolism Studies. Chirality 2: 269–274. 2083150 10.1002/chir.530020413

[10] Fell AF, Noctor TAG, Kaye B (1989) Invitro Metabolism Studies on Oxamniquine and Related-Compounds by Chiral Liquid-Chromatography. Journal of Pharmaceutical and Biomedical Analysis 7: 1743–1748. 2490562 10.1016/0731-7085(89)80189-x

[11] Kabsch W (2010) Xds. Acta Crystallographica Section D-Biological Crystallography 66: 125–132. 10.1107/S0907444909047337 PMC2815665 20124692

[12] Read RJ (1986) Improved Fourier Coefficients for Maps Using Phases from Partial Structures with Errors. Acta Crystallographica Section A 42: 140–149.

[13] Adams PD, Afonine PV, Bunkoczi G, Chen VB, Davis IW, et al (2010) PHENIX: a comprehensive Python-based system for macromolecular structure solution. Acta Crystallogr D Biol Crystallogr 66: 213–221. doi: 10.1107/S0907444909052925 20124702 10.1107/S0907444909052925 PMC2815670

[14] Emsley P, Lohkamp B, Scott WG, Cowtan K (2010) Features and development of Coot. Acta Crystallographica Section D-Biological Crystallography 66: 486–501. 10.1107/S0907444910007493 PMC2852313 20383002

[15] Schrodinger, LLC (2010) The PyMOL Molecular Graphics System, Version 1.3r1.

[16] Berman HM, Westbrook J, Feng Z, Gilliland G, Bhat TN, et al (2000) The Protein Data Bank. Nucleic Acids Res 28: 235–242. 10592235 10.1093/nar/28.1.235 PMC102472

[17] Webster P, Mansour TE, Bieber D (1989) Isolation of a Female-Specific, Highly Repeated Schistosoma-Mansoni DNA Probe and Its Use in an Assay of Cercarial Sex. Molecular and Biochemical Parasitology 36: 217–222. 2797060 10.1016/0166-6851(89)90169-2

[18] Delgado VS, Suarez DP, Cesari IM, Incani RN (1992) Experimental chemotherapy of Schistosoma mansoni with praziquantel and oxamniquine: differential effect of single or combined formulations of drugs on various strains and on both sexes of the parasite. Parasitol Res 78: 648–654. 1480600 10.1007/BF00931515

[19] Cross LC, Klyne W (1976) Report from Iupac Commission on Nomenclature of Organic-Chemistry—Rules for Nomenclature of Organic-Chemistry.E. Stereochemistry (Recommendations 1974). Pure and Applied Chemistry 45: 13–30.

