# Supplementary material for: Structural and Functional Characterization of the Enantiomers of the Antischistosomal Drug Oxamniquine
Source: PLoS Negl Trop Dis. 2015 Oct 20;9(10):e0004132. doi: 10.1371/journal.pntd.0004132 (PMC4618941; doi:10.1371/journal.pntd.0004132)

**Supplementary Figure 1.**

**Water structure in contact with oxamniquine enantiomers.** Superimposed structures of *R*- and *S*-OXA complex structures in green and yellow, respectively. Hydrogen bonding distances are indicated as dashed lines with distances in Å. Structurally equivalent water molecules preserved in both enantiomer complexes are shown as dark red spheres. Water molecules unique to the *R*-OXA complex are shown as green spheres and those unique to the *S*-OXA complex are shown as yellow spheres. Note: some structural elements have been removed for clarity.


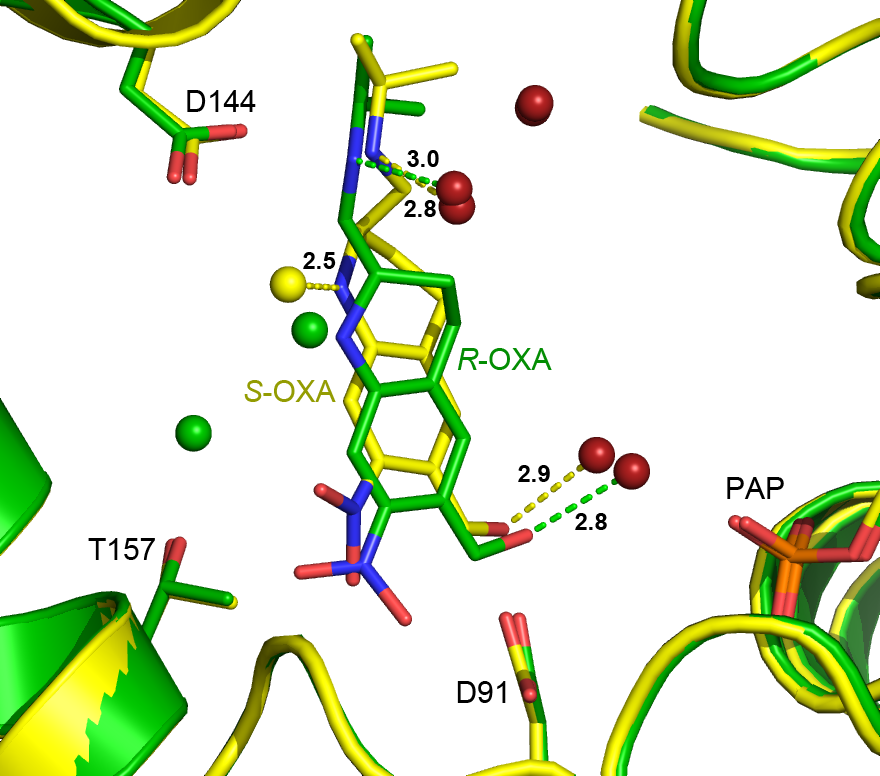

Supplement: S1 Fig — Superimposed structures of R- and S-OXA complex structures in green and yellow, respectively. (DOCX) [file pntd.0004132.s001.docx]
