# Supplementary material for: Structural and Functional Characterization of the Enantiomers of the Antischistosomal Drug Oxamniquine
Source: PLoS Negl Trop Dis. 2015 Oct 20;9(10):e0004132. doi: 10.1371/journal.pntd.0004132 (PMC4618941; doi:10.1371/journal.pntd.0004132)
Supplement: S2 Fig — Ten adult male worms per well. Three wells per drug. Treated for 45min with 40 μg/ml of OXA and 20 μg/ml of each enantiomer or DMSO equivalent. (DOCX) [file pntd.0004132.s002.docx]

**Supplementary Figure 2**

**Assay for efficacy of racemic OXA and its enantiomers**. Ten adult male worms per well. Three wells per drug. Treated for 45min with 40 μg/ml of OXA and 20 μg/ml of each enantiomer or DMSO equivalent.


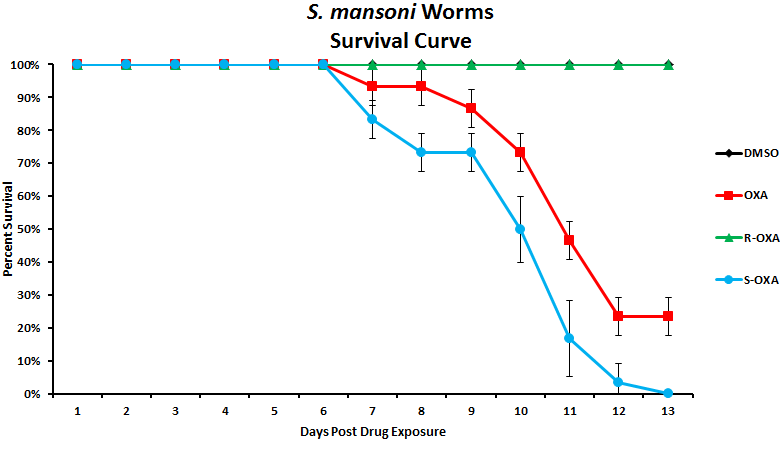


*Parasite maintenance, San Antonio*

*Schistosoma mansoni* LE strain is maintained in the laboratory in Golden Syrian hamsters as definitive hosts and *Biomphalaria glabrata* as the intermediate host. Patent hamsters (45 days post infection) are euthanized and the adult worms obtained by perfusion of the hepato-portal system [[1](#_ENREF_1)9].

*Drug assay, San Antonio*

The recovered worms were tested for drug sensitivity to racemic OXA and to both OXA enantiomers. Drug sensitivity was measured using a modified protocol [[2](#_ENREF_2)0]. Ten adult male worms were placed into each well of a 12-well microplate containing 3 mL of Dulbecco modified Minimum Eagle’s Medium (DMEM) supplemented with 20% newborn bovine serum, 100 U/mL penicillin, 100 µg/mL streptomycin and 0.5 µg/mL amphotericin B. Control and experimental groups were performed in triplicate. Adult worms were exposed to 40 µg/mL of OXA for 45 minutes followed by 3 washes with drug-free medium and incubated at 37ºC in an atmosphere of 5% CO_2_ for 12 days. Control groups were exposed only to the drug diluent dimethyl sulfoxide (DMSO). The parasites were then observed daily for 12 days under a stereomicroscope and the number of dead worms visually scored. Worms were defined as “dead” if they showed no movements and had acquired an opaque appearance. Drug sensitivity was scored as a binary trait. Parasites that remained alive for the 12 day period were classed as non-sensitive (>90% survival compared with controls). Parasites that showed high mortality by day 12 (<10% survival relative to controls) were classed as sensitive.
